# High Specific and Rapid Detection of Cannabidiol by Gold Nanoparticle-Based Paper Sensor

**DOI:** 10.3390/bios13110960

**Published:** 2023-10-28

**Authors:** Yufeng Sun, Dong Zhu, Ran Tao, Long Li, Bei Fan, Fengzhong Wang

**Affiliations:** Institute of Food Science and Technology, Chinese Academy of Agricultural Sciences, Beijing 100193, China; sunyufeng@caas.cn (Y.S.); zhudong0111@163.com (D.Z.); taoran@caas.cn (R.T.); lilong@caas.cn (L.L.)

**Keywords:** cannabidiol, immunochromatographic strip, monoclonal antibody, hapten, hemp

## Abstract

In order to facilitate monitoring of cannabidiol (CBD), we devised a gold immunochromatographic sensor based on a specific monoclonal antibody (mAb). To prepare the antigen, a novel hapten with CBD moiety and a linear carbon chain was employed. By utilizing hybridoma technology, a specific mAb was screened and identified that exhibited a 50% maximal inhibitory concentration against CBD ranging from 28.97 to 443.97 ng/mL. Extensive optimization led to the establishment of visual limits of detection for CBD, achieving a remarkable sensitivity of 8 μg/mL in the assay buffer. To showcase the accuracy and stability, an analysis of CBD-spiked wine, sparkling water, and sports drink was conducted. The recovery rates observed were as follows: 88.4–109.2% for wine, 89.9–107.8% for sparkling water, and 83.2–95.5% for sports drink. Furthermore, the coefficient of variation remained impressively low, less than 4.38% for wine, less than 2.07% for sparkling water, and less than 6.34% for sports drink. Importantly, the developed sensor exhibited no cross-reaction with tetrahydrocannabinol (THC). In conclusion, the proposed paper sensor, employing gold nanoparticles, offers a user-friendly and efficient approach for the precise, rapid, and dependable determination of CBD in products.

## 1. Introduction

**Figure 1:**
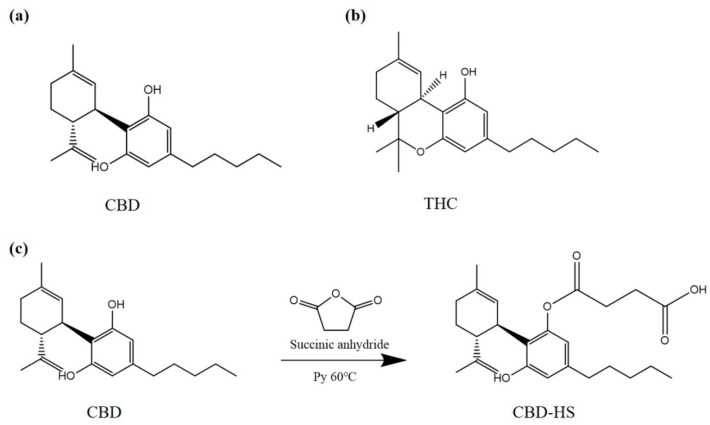
(**a**) CBD structure; (**b**) THC structure; (**c**) synthetic route of CBD hapten.

Hemp (*Cannabis sativa* L.) was first reported by Chinese Emperor Shen-nung Pen Ts’ao ching in the 28th century BCE [1]. It is valued for its fiber, seed, and active compounds. Cannabidiol (CBD, Figure 1a) is a non-psychoactive phytocannabinoid in hemp, which has the ability to regulate a wide range of physiological states and functions like memory, emotional state, and pain [2]. Public interest in CBD-derived products is growing. It has been explored as a potential agent for clinical use in various disorders, including cancer [3]. It also has been used in food, cosmetics, e-cigarettes, etc. [4]. For example, to acquire health functions such as anti-inflammatory, anti-aging, and immune regulation, CBD wine has been developed by adding CBD alone to wine or companying CBD with an emulsifier or other compositions and then adding to wine. Furthermore, hemp seed-derived products, such as snacks, sweets, and nutritional supplements, have been inferred containing CBD [5]. Tetrahydrocannabinol (THC, Figure 1b), a highly psychoactive compound considered as a prohibited substance, is the sister molecule of CBD. They are the major constituents of hemp, accounting for about 90% of cannabinoids. CBD accumulates during hemp growth. In recent years, CBD has demonstrated that it can be converted into THC under some conditions, such as acidic environment [6,7] and e-cigarettes [8]. Up until now, CBD safety for humans has not been clearly defined. In fact, many countries have strict regulations on the use of CBD. The Food and Drug Administration (FDA) closely scrutinizes CBD-derived products that could pose risks to consumers. Additionally, it defines that introducing food containing CBD into interstate commerce or marketing CBD products as, or in, dietary supplements is unlawful [9]. In China, it has become a consensus that CBD is not allowed in food. Considering the aforementioned information, it becomes crucial to formulate efficient techniques to detect CBD for its effective supervision.

Current CBD detection methods include high-performance liquid chromatography [10,11], high-performance liquid chromatography–mass spectrometry [12,13], gas chromatography [14], gas chromatography–mass spectrometry [15,16], and nuclear magnetic resonance spectroscopy [17]. Despite being sensitive and dependable, certain drawbacks render these techniques unsuitable for onsite detection. These disadvantages include the requirement for extensively trained personnel and costly equipment, as well as the complexity of sample pre-processing and the overall high expenses involved [18,19].

Immunoassays, which are time-saving, convenient, and cost-effective, have become extensively used for quality control in food industries [20,21,22,23,24], environmental pollutant analysis [25,26], and disease diagnosis [27,28]. Immunochromatographic assay (ICA) proves to be a better choice when it comes to swift detection compared with alternative types of immunoassays [29]. Nanoparticles (gold nanoparticles (GNPs)) can be used to visualize and amplify signals [30,31]. This method ensures affordability and eliminates the requirement for extensively trained personnel or expensive equipment. However, up until now, unlike the rapid emergence of ICA methods for THC detection [32], few ICAs have been developed for the determination of CBD [33].

In this research, we designed and synthesized a novel hapten and prepared a monoclonal antibody (mAb) that can specifically identify CBD. Based on this mAb, we developed a colloidal gold immunochromatographic assay (GICA) for detecting CBD using a series of optimization steps. Furthermore, an analysis of CBD-spiked wine, sparkling water, and sports drink was conducted to determine the accuracy and stability of the measurement. 

## 2. Materials and Methods

### 2.1. Reagents and Instruments

Cannabidiol (CBD) was obtained from Hancheng Yuanbeibei (Xian, China). Other chemical materials, such as succinic anhydride (HS), were obtained from Sinopharm (Beijing, China). Biomaterials, such as bovine serum albumin (BSA), were purchased from Kinbio (Shanghai, China). BALB/C female rats were obtained from Peking University. SP2/0-Ag14 tumor cells were obtained from Peking Union Cell Bank.

HPLC analyses were performed with 1220 infinityⅡLC (Agilent, Santa Clara, CA, USA). HRMS was carried out using Q Exactive Focus (Thermo Fisher, Waltham, MA, USA). ^1^H-NMR spectra were recorded by Bruker AV600 spectrometer (Bruker- Biospin AG, Fällanden, Swiss). MALDI-TOF was recorded with Bruker Autoflex Ⅲ (Bruker-Spectrospin AG, Karlsruhe, Germany). The T and C values were read by a hand-held reader (Huaan Magnech, Beijing, China).

### 2.2. Synthesis of Hapten

The hapten of CBD was designed by introducing the connecting arm and the active group for coupling macromolecules based on preserving the basic structure of CBD (Figure 1c). It was synthesized as previously reported [34]. Briefly, solution A was prepared by dissolving CBD (200 mg) in Py (3 mL), and solution B was prepared by dissolving HS (31.8 mg) in Py (3 mL). Then, solution B was added dropwise to solution A and mixed at 60 °C for 24 h, during which HS (31.8 mg) was added every 8 h. The mixture was vacuum-distilled and subsequently extracted. The residue was purified by column chromatography on silica gel using ethyl acetate–petroleum (60–90 °C) at a ratio of 1:3 as the eluent to afford hapten (CBD-HS). The hapten was characterized by HPLC, HRMS, and ^1^H-NMR.

### 2.3. Preparation of Antigen 

Immunogen CBD hapten BSA (CBD-HS-BSA) and coating antigen CBD hapten ovalbumin (CBD-HS-OVA) were produced by coupling CBD-HS with BSA and OVA via an active ester method [35]. Activation solution was prepared by dissolving CBD (2.7 mg), N-hydroxysuccinimide (NHS, 1.5 mg), and 1-ethyl-3-(3-dimethylaminopropyl) carbodiimide (EDC, 2.5 mg) in N, N-dimethylformamide (DMF, 0.5 mL) and stirring for 6 h at 25 °C. Then, the mixture was added slowly into BSA solution (20.0 mg of BSA added into 0.01 M PBS, 2 mL), stirred for 8 h, and subsequently dialyzed in PBS (pH 7.4) for 72 h at 4 °C to isolate CBD-HS-BSA. The same method was used to prepare CBD-HS-OVA. Both CBD-HS-BSA and CBD-HS-OVA were identified by MALDI-TOF. The coupling ratio (*CR_1_*) was calculated according to the following Equation (1):

(1) $${CR}_{1}\;\left(\%\right)={(M}_{p}-M_{std}/M_{h})\times 100$$

where *M_p_* is the molecular weight of CBD-HS-BSA or CBD-HS-OVA, *M_std_* is the molecular weight of BSA or OVA, and *M_h_* is the molecular weight of the CBD-HS.

### 2.4. Preparation of mAb

Seven BALB/c female mice (6–8 weeks of age) were fed for 14 days. The mice were immunized on the back and neck with immunogen CBD-HS-BSA (100 μg) emulsified with Freund’s complete adjuvant, followed by booster immunizations (100 μg of CBD-HS-BSA emulsified with Freund’s incomplete adjuvant) after 14 days. Five sequential boosters were performed at 14-day intervals. After each booster, the serum collected from the orbit of each mouse was tested for the antibody specificity by ic-ELISA. The mice with the highest potency and the best specificity to CBD were selected to be injected intraperitoneally with CBD-HS-BSA (50 μg) dissolved in normal saline (50 μL). The spleen cells of the mice were fused with SP2/0 cells by PEG 1500 after 5 days. Cell fusion was then performed [36]. The obtained cells were put into 96-well plates and cultured in a 5% CO_2_ incubator. The supernatant was authenticated 10 days later. The positive cell lines were selected to produce optimal monoclonal hybridoma cells. Finally, the objective cell was injected into the abdomen of BALB/c female mice to obtain ascites, which was subsequently purified 7 days later. mAb sensitivity was evaluated in ic-ELISA by measuring at half-maximum inhibitory concentration (IC_50_) [37], which was 50% inhibition concentration of CBD against CBD-HS-OVA combined with the antibody. mAb specificity was determined in ic-ELISA by measuring cross-reactivity (*CR*_2_), which was determined by the following Equation (2):

(2) $${CR}_{2}\;\left(\%\right)=(CBD\;{IC}_{50}/THC\;{IC}_{50})\times 100$$

### 2.5. Preparation of GNP–mAb Conjugate

#### 2.5.1. Preparation and Characterization of GNP

GNP was prepared as previously reported [38]. Briefly, chloroauric acid solution (1% in water, 5 mL) was added to ultrapure water (45 mL) and heated to boiling. Subsequently, trisodium citrate solution (1%, 0.6 mL) was quickly added. The mixture was reacted until it turned to a red-wine color and cooled to 25 °C to obtain GNP solution. The product was characterized using UV-vis spectrophotometer and transmission electron microscope (TEM).

#### 2.5.2. Determination of Optimum Amount of Potassium Carbonate

GNP solution (1 mL) was added to nine tubes, respectively, followed by 1–10 μL of potassium carbonate (K_2_CO_3_, 0.1 M), and mixed well. Then, mAb (1 mg/mL, 5 μL) was added to the mixtures, respectively. After reaction at 25 °C for 30 min, the minimum amount of K_2_CO_3_ with red-colored solution was recorded, which was the optimum amount of K_2_CO_3_. The result was verified by GICA strips.

#### 2.5.3. GNP–mAb Conjugate Preparation and Determination of Optimum Amount of mAb

In this experiment, a high-speed centrifugation method was used to optimize the amount of labeled antibody. Briefly, GNP solution (1 mL) was added to six tubes, respectively, followed by K_2_CO_3_ (2 μL), and mixed well. Subsequently, 2–8 μL of mAb (1 mg/mL) was added to the mixtures, respectively. After reaction at 25 °C for 10 min, BSA solution (3%, 50 μL) was added to close for 10 min, followed by centrifugation (4 °C, 8000 r/min) for 10 min. The residue was resuspended with colloidal gold complex solution (500 μL, 20 mM Tris pH 8.2, 0.1% Tween, 0.1% PEG, 5% trehalose, 5% sucrose, 0.2% BSA). The state of GNP–mAb solution was observed. The optimum amount of mAb was verified by GICA strips.

### 2.6. Preparation of GICA Strips

#### 2.6.1. Assembly of GICA Strips

**Figure 2:**
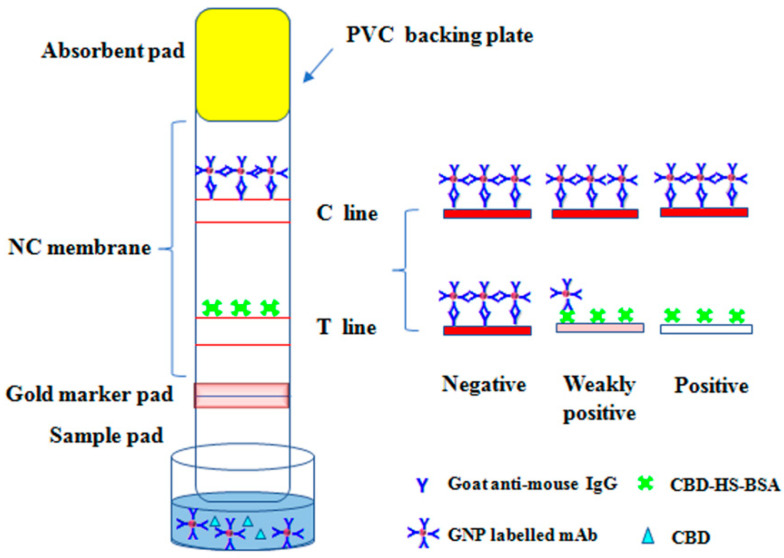
Schematic illustration and principle of the sensor.

GICA strips consisted of a sample pad, gold marker pad, absorbent pad, and nitrocellulose (NC) membrane, which were pasted on a polyvinyl chloride (PVC) bottom plate, as shown in Figure 2. GICA strips were assembled in such a way that the sample pad overlapped the gold marker pad by 1–2 mm, the gold marker pad overlapped the NC membrane by 1–2 mm, and the absorbent pad overlapped the NC membrane by 1–2 mm. Then, coating antigen CBD-HS-OVA and goat anti-mouse IgG antibody were utilized to form the T and C lines. After drying for 2 h at 37 °C, it could be cut to strips of 60 mm × 4 mm using an automatic chopping machine to obtain GICA strips.

#### 2.6.2. Optimization of Envelope Concentration of T and C Lines

In the GICA strips, the T line was enveloped with CBD-HS-OVA and the C line was enveloped with IgG. In order to obtain excellent GICA strips, different combinations of CBD-HS-OVA/goat anti-mouse IgG concentrations were selected and color performance was investigated to determine the optimum envelope concentration of the T line and the C line.

### 2.7. Analysis of Wine, Sparkling Water, and Sports Drink by GICA

Since hemp-related products containing CBD are unavailable due to strict regulations, common wine, sparkling water, and sports drink spiked with CBD material were used to assess the accuracy and stability of the GICA strips. Firstly, CBD standard solutions prepared with methanol were added to wine (1 mL), sparkling water (1 mL), and sports drink (1 mL), respectively, to obtain different “positive” samples (0, 5, 20, 30, 40 μg/mL). The “positive” samples were diluted with PBS solution (4 mL, 0.1 M, pH 7.0). The final solutions were then used for GICA analysis. Each group was repeated three times. For the GICA test, a 120 μL sample solution was added into a microplate containing GNP-labeled mAb, followed by insertion of a GICA strip. Due to capillarity action, the solution flowed from the sample pad to the absorbent pad. Six minutes later, the strip was removed and the color of the T line and the C line was observed. The T/C value was recorded using a portable reading instrument.

### 2.8. Statistical Analysis

All the tests were conducted using Origin (2019).

## 3. Results and Discussion

### 3.1. Synthesis and Characterization of Hapten and Antigen

It is known that the size of the immunogen is very important for obtaining high quality monoclonal antibodies [39]. The molecular weight of CBD is too low to induce a specific immune response. Therefore, CBD must be conjugated with a carrier protein to generate immunogenicity. In this work, the hapten of CBD was acquired by introducing the connecting arm and the active group for coupling macromolecules based on preserving the basic structure of CBD. And it was conjugated with BSA and OVA to afford the immunogen and the coating antigen, respectively. 

The purity of the CBD-HS was determined by HPLC, which was 93% (Figure S1a). It was also characterized by HRMS (Figure S1b) and ^1^H-NMR (Figure S1c). The results were as follows. HRMS [M + H]^+^: the theoretical value was 415.24790 and the measured value was 415.24780. ^1^H-NMR (300 MHz, DMSO-d6): 0.85 (t, *J* = 9.0 Hz, 3H), 1.15–1.27 (m, 6H), 1.59–1.66 (m, 9H), 1.99 (S, 2H), 2.40 (t, *J* = 6.0 Hz, 4H), 2.69 (q, *J* = 8.0 Hz, 2H), 4.03 (q, *J* = 21.0 Hz, 1H), 4.42 (S, 2H), 5.01 (S, 1H), 6.20 (S, 1H), 6.45 (S, 1H), 9.30 (S, 1H).

CBD-HS-BSA and CBD-HS-OVA were identified by MALDI-TOF (Figure S2). The molecular weights of CBD-HS-BSA and CBD-HS-OVA were 72026.339 and 46198.120, respectively. CBD-HS-BSA had a coupling ratio of 12:1, indicating that it could be used as an immune antigen. CBD-HS-OVA had a coupling ratio of 3:1, indicating that it could be used as an encapsulated antigen.

### 3.2. Characterization of mAb

**Figure 3:**
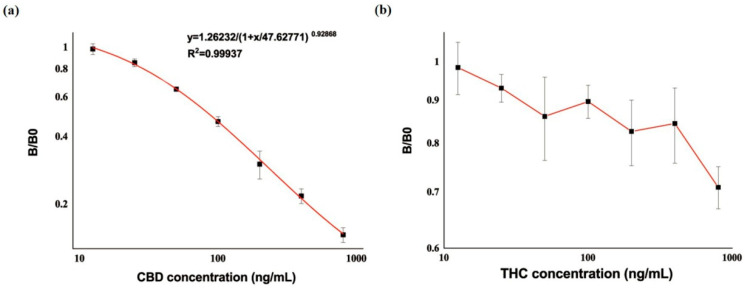
The standard curve of mAb in ic-ELISA. (**a**) For CBD; (**b**) for THC. A log scale was used.

A monoclonal cell CBD-12A1 producing mAb against CBD was obtained in this work. To investigate the sensitivity of mAb (anti-CBD-12A1), a standard curve was established by applying ic-ELISA. The typical calibration curve illustrated by plotting absorbance ratio (B/B0) against CBD concentration is shown in Figure 3a. Anti-CBD-12A1 showed an IC_50_ value of 87.43 ng/mL for CBD, and the range of detection was 28.97–443.97 ng/mL, with a calculated limit of detection (cLOD) of 28.97 ng/mL. Here, the concentration of 20% inhibition (IC_20_) of B/B0 was defined as cLOD. THC, an analogue with a similar structure to CBD, was selected to determine the specificity of anti-CBD-12A1. As shown in Figure 3b, there was no cross-reaction rate of anti-CBD-12A1 for THC, indicating an exhibited excellent specificity. In summary, anti-CBD-12A1 was a satisfactory monoclonal antibody with good sensitivity and specificity.

### 3.3. Preparation of GNP–mAb Conjugate 

#### 3.3.1. Characterization of GNP

**Figure 4:**
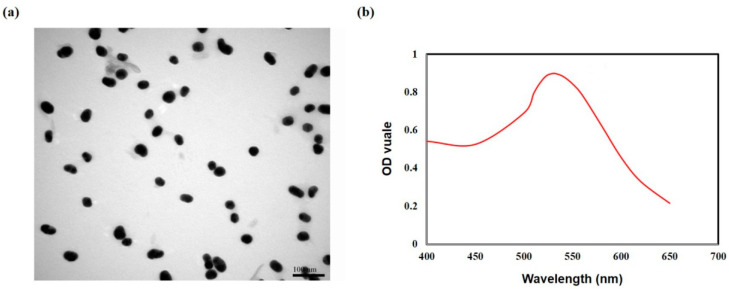
(**a**) TEM images of GNP solution; (**b**) UV-vis absorption spectrum of GNP solution.

In this work, GNP was prepared by the classical citrate reduction method. UV–vis spectrometry and TEM were utilized to characterize the GNP. As shown in Figure 4, GNP was well-distributed and appeared uniform (approximately 40 nm in diameter). UV–vis spectrometric analysis of the GNP showed an absorption peak close to 530 nm. It was reported that GNPs of 10–40 nm in diameter were optimal for ICA analysis because of their acceptable color development and stability [40]. Our pre-experiment confirmed that GNP with a diameter of 40 nm was stable and the produced wine color was suitable for ICA analysis. In solution, the prepared GNPs were spherical in shape, with smooth and complete edges. The certainty of their morphology is of great significance for their performance, such as surface effects, optical properties, and electrical properties, which will play an important role in the stability of detection [41].

#### 3.3.2. Bioconjugation of GNP with mAb

**Figure 5:**
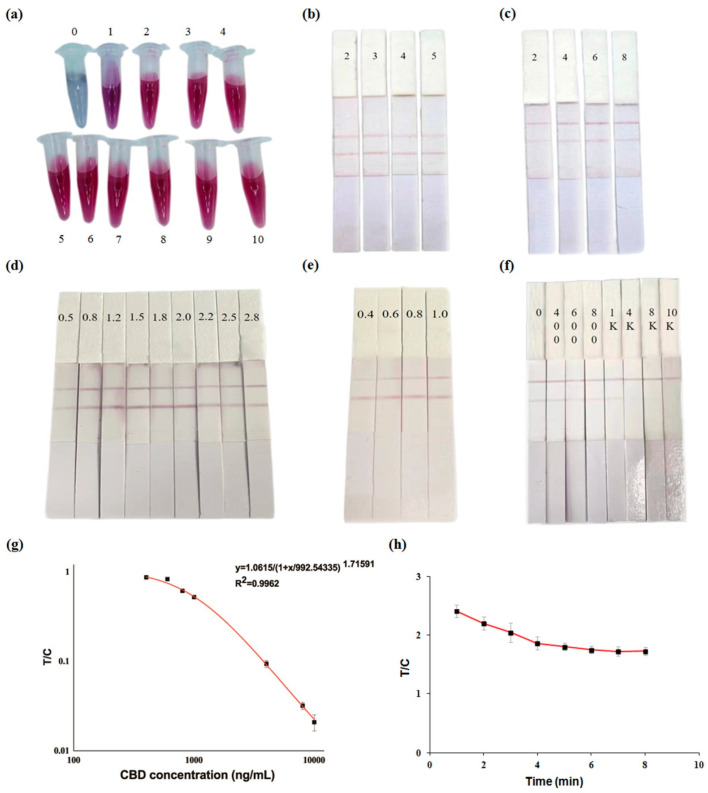
Optimization of important factors affecting GNP–mAb conjugate and GICA strip; performance of GICA strip. (**a**) K_2_CO_3_ usage (0, 1, 2, 3, 4, 5, 6, 7, 8, 9, 10 μL); (**b**) K_2_CO_3_ usage (2, 3, 4, 5 μL); (**c**) mAb usage (2, 4, 6, 8 μL); (**d**) envelope concentration of T line (0.5, 0.8, 1.2, 1.5, 1.8, 2.0, 2.2, 2.5, 2.8 mg/mL); (**e**) envelope concentration of C line (0.4, 0.6, 0.8, 1.0 mg/mL); (**f**) sensitivity of GICA strip for CBD (0, 400, 600, 800, 1000, 4000, 8000, 10,000 ng/mL); (**g**) standard curve of GICA strip for CBD standard solution; (**h**) determination of color development stability time of GICA strip. The T/C (ratio of values of T line and C line) value was obtained using a hand-held strip scan reader. A log scale was used in (**g**).

Colloidal GNP is bound to mAb mainly by utilizing electrostatic adsorption under alkaline conditions [18]. The pH of the system is one main factor influencing the stability of the GNP–mAb conjugate. Thus, the amount of K_2_CO_3_ used needs to be optimized. In this work, 0.01 M K_2_CO_3_ was used. As seen in Figure 5a, when the amount of K_2_CO_3_ in GNP solution was lower than 2 μL, the color of the solution turned gray or grey/purple and the particles began to cluster. When the amount of K_2_CO_3_ was higher than or equaled 2 μL, the particles were well-distributed. Excess K_2_CO_3_ could have influenced the conjugation of GNPs and mAb, and the solution color was caused to retain red. Then, 2–5 μL K_2_CO_3_ was selected to make the GNP–mAb conjugate used for preparing GICA strips to verify their effect. It was found that the color of the T and C lines did not change significantly (Figure 5b). Therefore, 2 μL of K_2_CO_3_ (0.01 M) was determined as the optimum amount for the labeling of mAb with GNP.

The amount of antibody was another factor affecting the conjugation reaction. In this work, the amount of mAb used was also optimized. As shown in Figure 5c, the increase in mAb addition led to the T line becoming darker. When 6 μL of mAb (1 mg/mL) was added, the T line color became maximally dark, indicating GNP–mAb conjugate saturation. Excess mAb may affect the conjugation activity because of aggregation [42]. Therefore, 6 μL of mAb (1 mg/mL) was found as the optimum amount for the labeling of mAb with GNP.

### 3.4. Preparation of GICA Strips

#### 3.4.1. Optimization of Envelope Concentration of T and C Lines

The optimum envelope concentration of the T and C lines was obtained by the control variable method. First, the envelope concentration of the C line was set to 1 mg/mL, and the envelope concentration of the T line was set to 0.5, 0.8, 1.2, 1.5, 1.8, 2.0, 2.2, 2.5, 2.8 mg/mL. As shown in Figure 5d, the increase in envelope concentration of the T line led to a darker T line, and when the concentration was 2 mg/mL, the T line color became maximally dark. When the concentration was higher than 2 mg/mL, the T line color gradually lightened. Thus, 2 mg/mL was determined as the optimum envelope concentration of the T line. Then, the envelope concentration of the T line was set to 2 mg/mL, and the envelope concentration of the C line was set to 0.4, 0.6, 0.8, 1.0 mg/mL. As shown in Figure 5e, the increase in envelope concentration of the C line gave a darker C line. When the concentration was 0.8 mg/mL, the C line color was close to that of the T line. Thus, 0.8 mg/mL was found as the optimum envelope concentration of the C line.

#### 3.4.2. Sensitivity and Specificity of GICA Strips

GICA strips were prepared based on the above optimized conditions. Their sensitivity was investigated by detecting CBD standard solution with increasing concentration (0, 400, 600, 800, 1000, 4000, 8000, 10,000 ng/mL). As can be seen in Figure 5f, CBD can be completely suppressed at 8000 ng /mL, which was the visual limit of detection (vLOD) of GICA strips for CBD. As shown in Figure 5g, the GICA strip showed an IC_50_ value of 1065 ng/mL for CBD, and the range of detection was 518–2342 ng/mL. The specificity of the GICA strip was investigated by detecting the THC standard solution with gradient concentration (0, 400, 600, 800, 1000, 4000, 8000, 10,000 ng/mL). The result showed that the GICA strips displayed negative to THC standard solution of any concentration (Figure S3), indicating an excellent specificity.

#### 3.4.3. Color Development Stability Time of GICA Strips

Color development stability time of the GICA strips was evaluated by reading the T, C, and T/C values of each strip at different times (1–8 min) with a hand-held strip reader. The color development of the GICA strips changed significantly between 1 and 4 min after chromatographic analysis, and the degree of change slowed down after 4 min, while the color development was basically stable at 6 min (Figure 5h). Therefore, the test result could be obtained within 6 min after using the GICA strip in the actual sample detection.

### 3.5. Analysis of Wine, Sparkling Water, and Sports Drink by GICA

The common wine, sparkling water, and sports drink purchased from the local supermarket were CBD-negative. The accuracy and the stability of the GICA strips were showcased based on the sample recovery experiment due to the unavailability of hemp-related products containing CBD in the local market. Given that different solvents can influence the antibody–antigen binding reaction [18], PBS solution was chosen to dilute CBD-spiked wine, sparkling water, and sports drink. From the original strip images (Figure S4), it can be seen that, as the CBD concentration increased in the spiked samples, the T lines of the GICA strips showed a lighter color. The spiked recovery ranged from 88.4 ± 3.0% to 109.2 ± 4.8%, with the coefficient of variation ranging from 0.43% to 4.38% (Table 1). The results indicated that the GICA strips exhibited good accuracy and stability and the reproducibility of the system was high.

## 4. Conclusions

In this study, we devised a GICA strip for the rapid detection of CBD based on GNP and specific mAb. Our results demonstrated its good performance for CBD detection in spiked wine, sparkling water, and sports drink. The recovery test verified the accuracy and reliability of the GICA strip. Moreover, it is user-friendly and the test can be completed within 6 min. In summary, the GICA strip can provide an efficient approach for the precise, rapid, and dependable determination of CBD in products. However, there are some limitations in this work. For example, the vLOD of the GICA strip for CBD detection is high. Future study should develop more excellent GICA strips by optimizing antibody labeling materials. Meanwhile, the combination of biosensors with other technologies such as machine learning algorithms [43] and stable isotope analysis [44] should be given attention.

## Figures and Tables

**Table 1:** Analysis of CBD in spiked wine, sparkling water, and sports drink sample by GICA (n = 3).

Sample	Added Level (μg/mL)	Found Value (μg/mL)	Recovery Rate (%)	Coefficient of Variation (%)
Wine	5	5.46	109.2 ± 4.8	4.38
	20	18.62	93.1 ± 4.1	1.10
	30	27.33	91.1 ± 3.8	0.70
	40	35.36	88.4 ± 3.0	0.43
Sparkling water	5	5.39	107.8 ± 2.2	2.07
	20	18.79	94.0 ± 5.2	1.38
	30	28.15	93.8 ± 7.2	1.27
	40	35.97	89.9 ± 8.1	1.12
Sports drink	5	4.77	95.5 ± 6.1	6.34
	20	16.65	83.2 ± 8.2	2.47
	30	26.93	89.8 ± 7.2	1.33
	40	34.72	86.8 ± 6.1	0.88

## Data Availability

This article is a free-access publication.

## Supplementary Materials

The following supporting information can be downloaded at: https://www.mdpi.com/article/10.3390/bios13110960/s1, Figure S1: liquid chromatogram, HRMS, ^1^N-NMR spectra of CBD hapten; Figure S2: MALDI TOF spectra of BSA, CBD-HS-BSA, OVA, and CBD-HS-OVA; Figure S3: specificity of GICA strip for THC; Figure S4: strip images of CBD in spiked wine, sparkling water, and sports drink samples.
