# Supplementary material for: High Specific and Rapid Detection of Cannabidiol by Gold Nanoparticle-Based Paper Sensor
Source: Biosensors (Basel). 2023 Oct 28;13(11):960. doi: 10.3390/bios13110960 (PMC10669437; doi:10.3390/bios13110960)
Supplement: Supplementary file 1 [file biosensors-13-00960-s001.zip › biosensors-2654041-supplementary.pdf]

biosensors

MDPI

Supplementary Material

High Specific and Rapid Detection of Cannabidiol by Gold Nanoparticle-Based Paper Sensor

Yufeng Sun †, Dong Zhu †, Ran Tao, Long Li, Bei Fan * and Fengzhong Wang *

Institute of Food Science and Technology, Chinese Academy of Agricultural Sciences, Beijing 100193, China; sunyufeng@caas.cn (Y.S.); zhudong0111@163.com (D.Z.); taoran@caas.cn (R.T.); lilong@caas.cn (L.L.)

* Correspondence: fanbei@caas.cn (B.F.); wangfengzhong@caas.cn (F.W.)

† These authors contributed equally to this work.

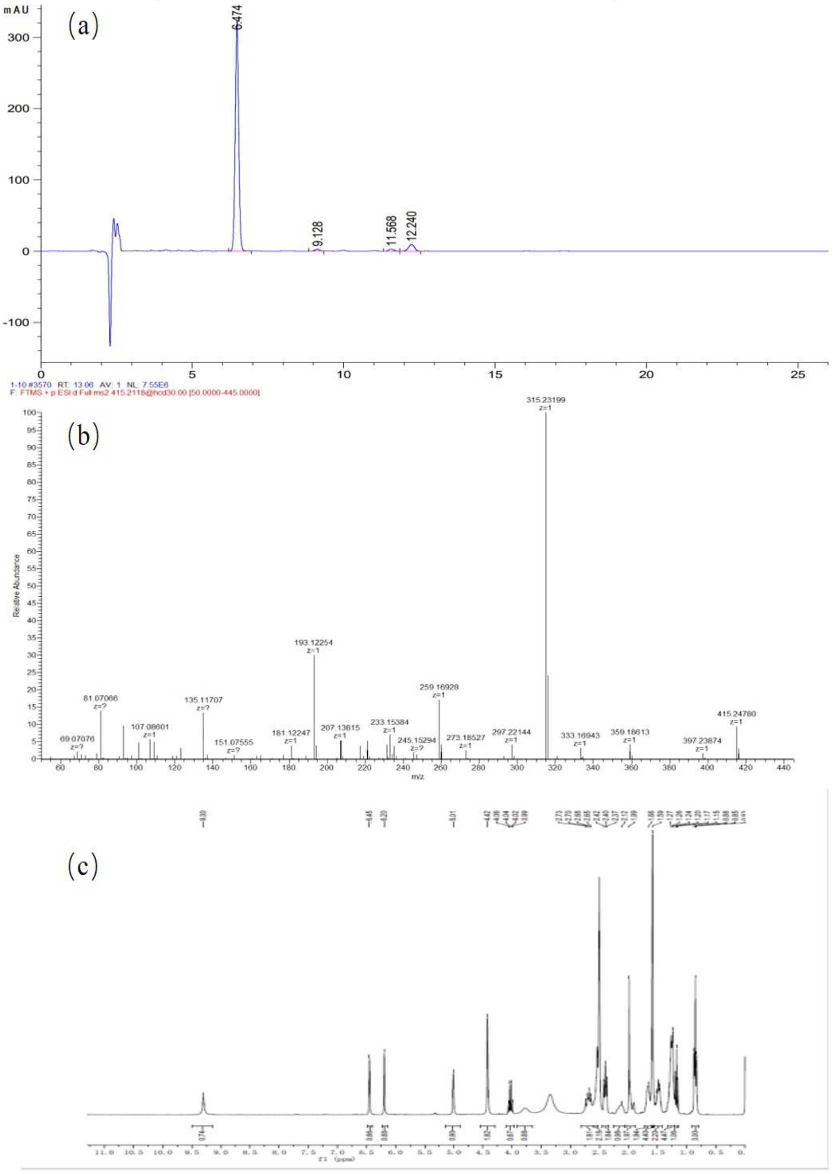

Figure S1. (a) Liquid chromatogram of CBD hapten; (b) HRMS of CBD hapten; (c) [1]N-NMR spectra of CBD hapten.

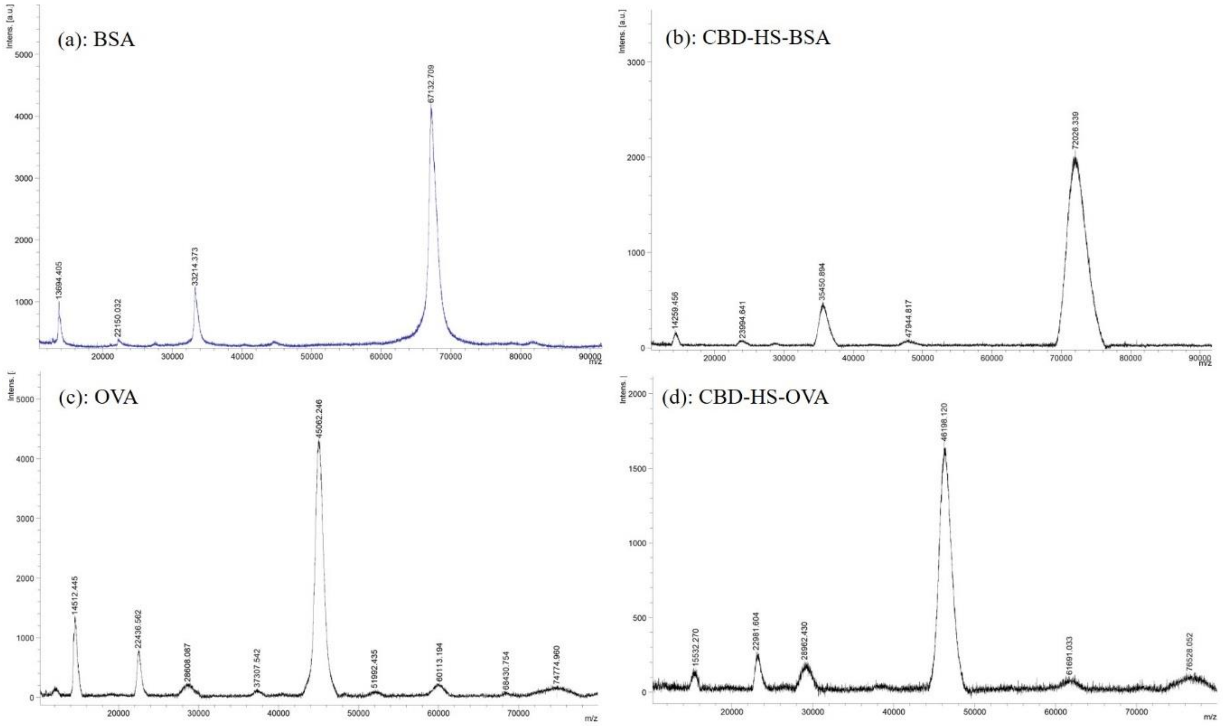

Figure S2. MALDI TOF spectra of (a) BSA, (b) CBD-HS-BSA, (c) OVA, and (d) CBD-HS-OVA.

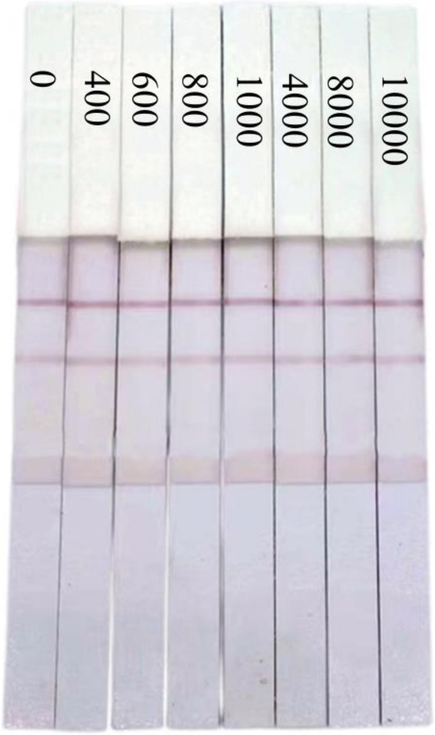

Figure S3. Specificity of GICA strip for THC (0, 400, 600, 800, 1000, 4000, 8000, 10000 ng/mL).

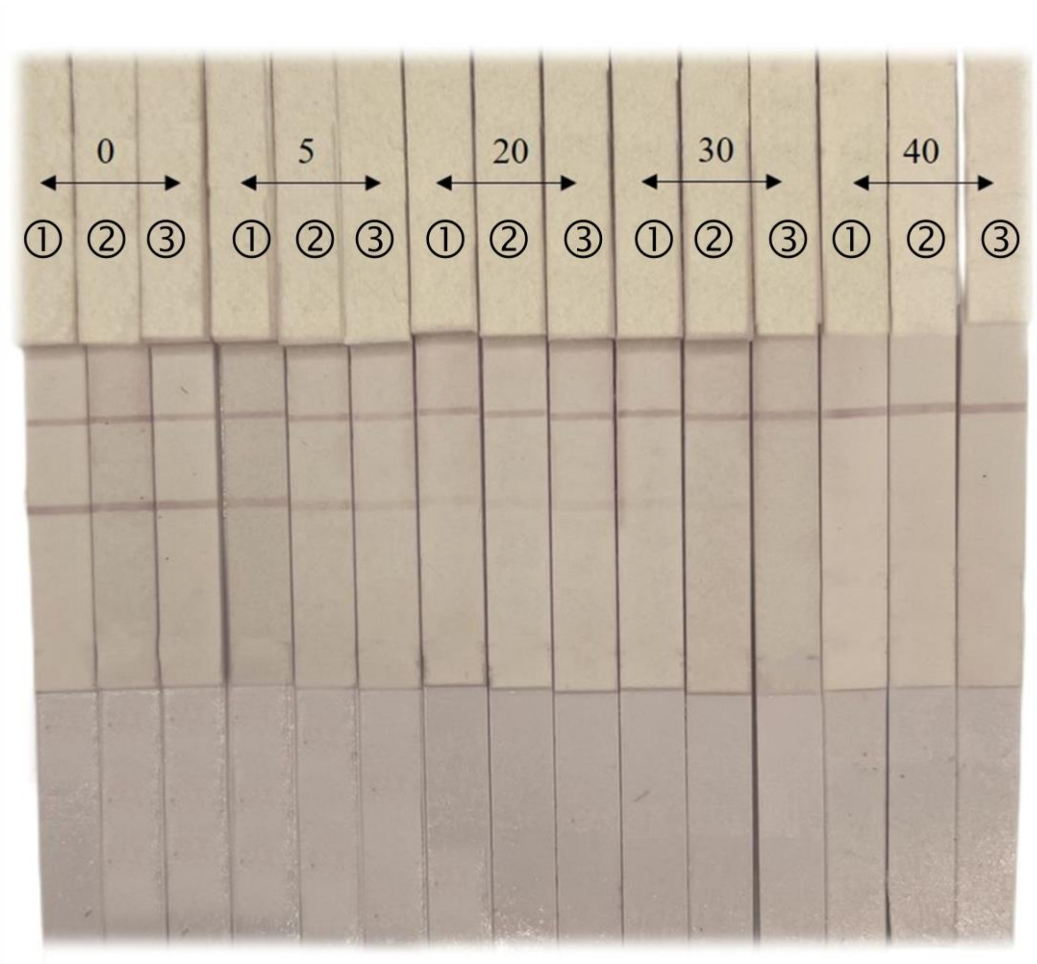

Figure S4. Strip images of CBD in spiked wine (①), sparking water (②) and sports drink (③) samples (0, 5, 20, 30, 40 μg/mL).
